# Stabilization of nanosized titanium dioxide by cyclodextrin polymers and its photocatalytic effect on the degradation of wastewater pollutants

**DOI:** 10.3762/bjoc.12.286

**Published:** 2016-12-28

**Authors:** Tamás Zoltán Agócs, István Puskás, Erzsébet Varga, Mónika Molnár, Éva Fenyvesi

**Affiliations:** 1 CycloLab Cyclodextrin R&D Laboratory Ltd, Illatos út 7, Budapest, 1097, Hungary; 2 Department of Applied Biotechnology and Food Science, Budapest University of Technology and Economics, Szent Gellért tér 4, Budapest, 1111, Hungary

**Keywords:** carboxymethyl β-cyclodextrin polymer, colloid stability, ibuprofen, methylene blue, nanoTiO_2_, synergetic effect, wastewater treatment

## Abstract

Advanced oxidation processes (AOPs) are considered highly competitive water treatment technologies for the removal of organic pollutants. Among AOP techniques, photocatalysis has recently been the most widely studied. Our aims were to investigate how the dispersion of nanosized titanium dioxide (nanoTiO_2_) applied in photodegradation-based procedures can be stabilized with cyclodextrins in order to obtain a new, more efficient photocatalyst for the purification of waters polluted by xenobiotics applying UV irradiation. During our work, on the one hand, we studied the behavior and stability of nanoTiO_2_ in cyclodextrin solutions. On the other hand, we used various monomer and polymer cyclodextrin derivatives, and assessed the options for nanoTiO_2_ stabilization in the presence of various salts and tap water on the basis of turbidity tests. The physical stability of nanoTiO_2_ dispersions is diminished in the presence of the salts found in tap water (and occurring also in surface waters and ground water) and they are precipitated immediately. This colloidal instability can be improved by cyclodextrin derivatives. Based on the results of our studies we have selected carboxymethyl β-cyclodextrin polymer (CMBCD-P) for stabilization of nanoTiO_2_ dispersions. The photocatalytic degradation of methylene blue and ibuprofen as model organic pollutants in various media (distilled water, NaCl solution and tap water) has been studied using nanoTiO_2_ as catalyst stabilized by CMBCD-P. CMBCD-P itself showed a catalytic effect on the UV degradation of methylene blue. In addition to enhancing the colloid stability of nanoTiO_2_ CMBCD-P showed also synergistic effects in catalyzing the photodecomposition process of the dye. On the other hand, ibuprofen as a model pharmaceutical, a pollutant of emerging concern (EP), was protected by CMBCD-P against the photocatalytic degradation showing that inclusion complex formation can result in opposite effects depending on the structure of the host–guest complex.

## Introduction

The wastewater purification and reuse are key challenges of our society. Globally 330 km^3^year^−1^ municipal wastewater is produced [1]. According to the European Investment Bank, by 2025, 800 million people will be living in regions in shortage of drinking water [2]. The recently recognized harmful xenobiotics (contaminants of emerging concern, such as pharmaceutical residues, personal care products, industrial additives) impose a high risk on the environment and also on human because they usually do not degrade in nature, appear in surface waters and groundwater, and may accumulate in living organisms [3–4]. From time to time, these microcontaminants can also be detected in tap water and may pose a risk for human, too [5]. These microcontaminants are xenobiotics for the microbes used in the secondary treatment (after sedimentation, which is the primary treatment) of wastewater, so these compounds are not efficiently eliminated with the conventional technologies from the effluent. To avoid the contamination of the receiving environment (sea, river, lake, wetlands, ground, etc.) it is essential that efficient and economical treatment procedure(s) are elaborated for the removal of such microcontaminants. Various sorbents containing cyclodextrin (CD) have been developed aiming at the removal of pharmaceutical residues, pesticides and other endocrine disrupting compounds from purified wastewater [6–7]. CDs are cyclic oligosaccharides consisting of 6–8 glucose units (α-, β- and γ-CD) primarily used in the pharmaceutical, cosmetic and household chemical industry [8]. They form non-covalent inclusion complexes with a great number of the organic contaminants in soil (petroleum hydrocarbons, polycyclic aromatic hydrocarbons, etc.) and microcontaminants occurring in water (pharmaceutical and cosmetic active agents, pesticides, etc.). The CD derivatives, which are well soluble in water, such as hydroxypropyl and methylated CDs, can enhance desorption of the contaminants from the soil and are useful in soil remediation technologies (e.g., in situ/ex situ microbial degradation and chemical oxidation of contaminants in soil) [9–10]. Immobilizing CDs either by crosslinking or by coupling to the surface of natural or synthetic polymers CD-based sorbents are obtained in the form of beads, nanosponges, microfibers, etc., which are able to remove microcontaminants from purified wastewater [11–17] and can be also used as samplers [12]. Further methods applying CDs for wastewater purification include CD-intensified biodegradation of contaminants [18], and oxidation of micropollutants adsorbed on cyclodextrin polymer (CDP) using KMnO_4_ [19].

Advanced oxidation processes (AOPs) based on in situ generation of highly reactive species can mineralize organic contaminants into relatively harmless compounds. Research activities have been recently focused on photocatalysis belonging to these AOP techniques [20–24]. The photodegradation is a technology utilizing the energy of light for decomposition of the contaminants. CD can catalyse or inhibit the photodecomposition of a compound depending on the position of the light-sensitive bonds of the included compound. For instance, photodegradation of bisphenol A was enhanced in aqueous solutions containing β-CD [25], while the photodecomposition of pesticides of similar structure (parathion and paraoxon) was inhibited or catalyzed, respectively, by β-CD [26]. The complex formation can either protect the drug from the effect of light or accelerate the decomposition [27–28].

Recently, various photocatalysts modified by CDs have been described. For instance, reduced graphene oxide/β-CD/titanium dioxide showed enhanced removal of phenol and Cr(VI) [29], graphene nanosheets with self-assembled nanolayer of TiO_2_ stabilized by β-CD resulted in improved photodegradation of methylene blue [24], and CD-functionalized Fe_3_O_4_/TiO_2_ was efficient catalyst in photodecomposition of endocrine disrupting compounds, such as bisphenol A and dibutyl phthalate [30].

**Figure 1:**
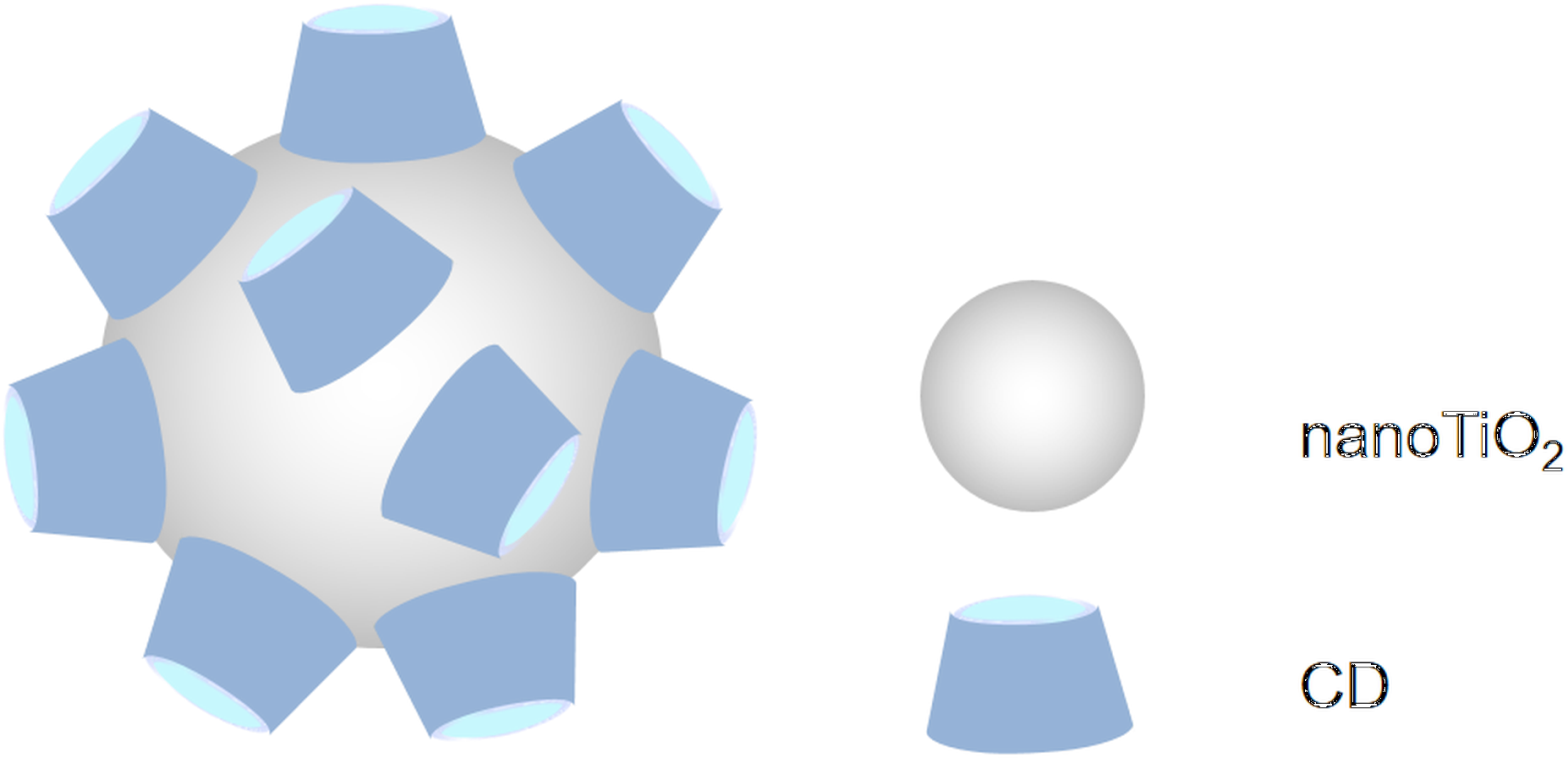
Adsorption of β-CD on the surface of nanoTiO_2_ [37].

Producing strong oxidizing radicals (hydroxyl and superoxide radical ions) titanium dioxide is a widely used catalyst for photodecomposition of various organic pollutants [31]. The photocatalytic reactions take place on the surface of the catalyst on the effect of solar light or of artificial UV light irradiation. In the practice, TiO_2_ is immobilized on a surface, e.g., glass wool mats or ceramic plates and a thin layer of waste water is treated by solar light to achieve the decomposition of the organic pollutants [32]. Another way of enhancing the surface is the application of nanoparticles (nanoTiO_2_). Such nanosized particles can readily aggregate forming larger agglomerates of reduced surface and reduced catalytic activity. The aggregation is a slow process in distilled water, but happens instantly in the presence of salts, e.g., in tap water, surface waters and waste waters. β-CD can be used for stabilization of colloidal TiO_2_ systems [33] – at least in distilled water – as it is adsorbed on the surface of the nanoparticles (with its wider secondary side, Figure 1) [34]. In addition to the improvement of the colloidal stability, also the efficiency of the photocatalytic performance of nanoTiO_2_ particles can be enhanced by adsorption of β-CD. The latter plays electron-donating and hole-capturing roles when linked to nanoTiO_2_ colloids leading to restriction of charge–hole recombination [35]. The efficiency is further improved by keeping the ligands close to the surface of nanoTiO_2_ via inclusion complexation. In other studies the presence of CDs caused a delay in the photocatalytic degradation of toluene [36].

Although several operating parameters (concentration of pollutants, pH, irradiation time etc.) have already been investigated most of the experiments published so far were performed only in distilled water [23,38–40]. In the present work we aimed at finding a cyclodextrin derivative which effectively protects nanoTiO_2_ in aqueous media against the precipitating effect of different salts in tap water. We used turbidity and light scattering measurements for studying the aggregation behavior of nanoTiO_2_. In a second step, the photocatalytic properties of the stabilized nanoTiO_2_ dispersions have been studied for degradation of some model organic pollutants (methylene blue and ibuprofen) under UV-A light irradiation.

## Results and Discussion

### Selection of the CD derivative for stabilizing nanoTiO_2_ dispersions

In aqueous dispersions, in the presence of salts the nanoparticles of TiO_2_ start to aggregate and form larger particles enhancing the turbidity of dispersion. The turbidity (the haziness of the dispersion caused by particles invisible for the naked eye) was measured to monitor the aggregation of the particles of nanoTiO_2_. Various monomer and polymer CD derivatives were studied how they influence the stability of nanoTiO_2_ in the presence of NaCl (0.1%) by recording the turbidity. We used neutral and charged CD derivatives, such as non-ionic 2-hydroxypropyl-β-cyclodextrin monomer (M) and polymer (P) (HPBCD-M/P), anionic carboxymethyl-β-cyclodextrin monomer and polymer (CMBCD-M/P) and quaternary ammonium β-cyclodextrin polymer (QABCD-P) in a concentration of 1% (50 mg/5 mL). The polymers were prepared by crosslinking the proper monomers with epichlorohydrin and contained 4–200 β-CD units. None of the monomers could hinder the aggregation of nanoTiO_2_ in 0.1% NaCl solution while the polymers showed stabilizing effect (Table 1).

**Table 1:** Turbidity of 0.02% nanoTiO_2_ dispersions in the presence of 1% CD polymers in distilled water and in 0.1% NaCl solution at 120 min related to the initial turbidity (100%).

	No CD	HPBCD-P	CMBCD-P	QABCD-P

Distilled water	100 ± 2%	104 ± 2%	101 ± 2%	101 ± 2%
0.1% NaCl solution	209 ± 5%	107 ± 2%	101 ± 2%	103 ± 2%

The effects of the polymer concentration (1% and 5%) and its average molecular weight (90 kDa, 200 kDa, 300 kDa) on the colloidal stability of the nanoTiO_2_ dispersion were also studied using the neutral HPBCD-P. It was clearly shown that the stabilization effect was not influenced by the average molecular weight and there was no remarkable difference between the samples with 1% and 5% polymer concentration (the turbidity in salt solution changed to 102–107% and 102–105% related to the initial after 120 min, respectively. Results are not presented here.)

**Figure 2:**
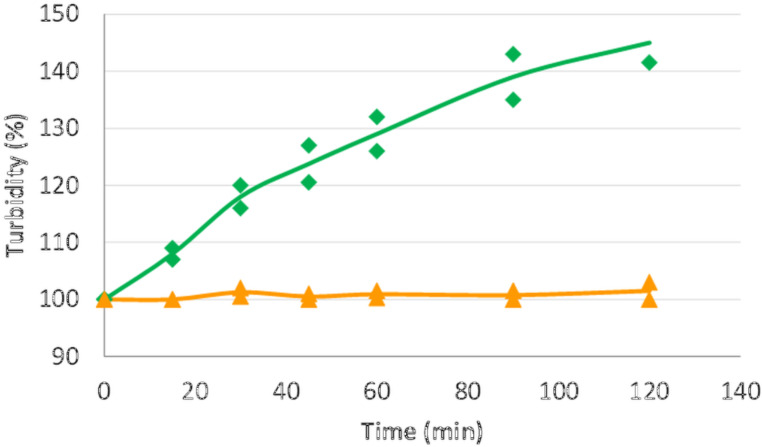
Turbidity of nanoTiO_2_ dispersion (0.02%) in the presence of 1% HPBCD-P (green diamond) and 1% CMBCD-P (yellow triangle) in tap water.

After diluting nanoTiO_2_ dispersions with tap water immediate precipitation was observed. Among the CD polymers studied the QABCD-P could not hinder this precipitation, the HPBCD-P slowed down the precipitation process, while in the presence of CMBCD-P no precipitation occurred in 120 min (Figure 2).

As the lowest enhancement in turbidity both in NaCl solution and in tap water was obtained with the polymer of the carboxymethyl β-CD derivative (CMBCD-P), this material was selected for further studies.

The colloidal stability of nanoTiO_2_ dispersions in various salt solutions was examined in the presence of some selected ions typical in tap water. Without the CD polymer additive the nanoTiO_2_ was immediately precipitated in the solution of 0.1% Na_2_SO_4_, and a fast enhancement of the turbidity was observed in the presence of the other salts: Ca/MgCl_2_ and Na_2_CO_3_. The dispersion was stable at as low concentration as 1% CMBCD-P and the turbidity remained <105% within 120 min of observation (Table 2).

**Table 2:** The effect of salts (0.1%) on the turbidity of nanoTiO_2_ aqueous dispersions (0.02%) in the presence and absence of CMBCD-P (1%)^a^.

	Relative turbidity (%)				
	
	NaCl	CaCl_2_	MgCl_2_	Na_2_CO_3_	Na_2_SO_4_

No CD	209%	>200%*	>200%**	220%*	immediate precipitation
CMBCD-P	100.1%	100.2%	100.1%	100.4%	100.0%

^a^Turbidity after 120 min (*20 min, ** 60 min) related to the initial value.

The stabilizing effect of the CD-based polymers especially of CMBCD-P can be attributed to steric effects caused by the adsorption of the polymer on the surface of nanoTiO_2_ particles. It forms a layer, which inhibits their aggregation. The negative charge contributes to the stabilizing effect by i) electrostatic attraction of the sorbed layer to the positively charged holes on the nanoTiO_2_ particles and ii) repulsion between the particles covered by the polymer layer.

### Aggregation studies

Based on particle size measurements the aggregation behavior of nanoTiO_2_ in the presence and absence of polymer was compared in different media. We studied the effect of NaCl and tap water.

**Figure 3:**
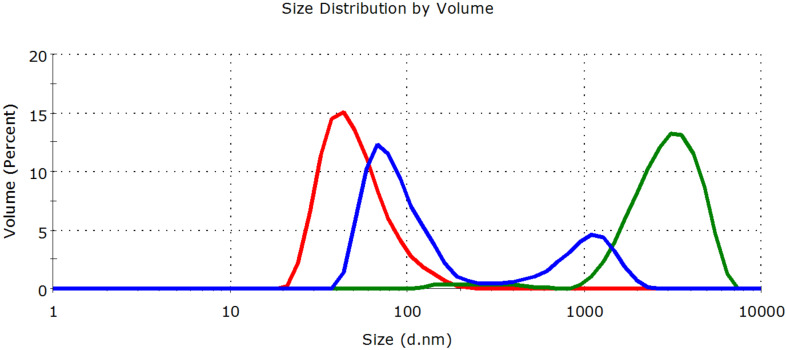
Aggregation effect of 0.1% NaCl on 0.02% nanoTiO_2_ dispersion in the absence (green curve) and presence of CMBCD-P polymer (blue curve). The red curve shows the particle size distribution in the absence of destabilizing ions.

**Figure 4:**
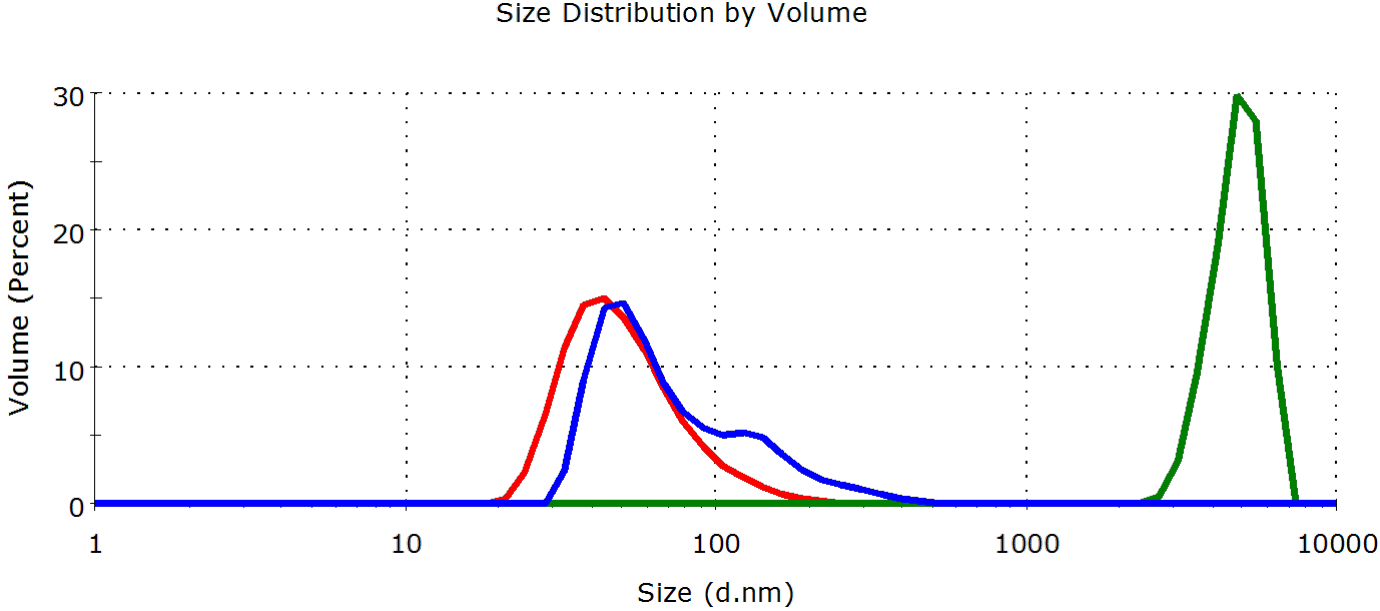
Aggregation effect of tap water on 0.02% nanoTiO2 dispersion in the absence (green curve) and presence of CMBCD-P polymer (blue curve). The red curve shows the particle size distribution in the absence of destabilizing ions.

The applied nanoTiO_2_ consists of crystallites of 10–20 nm diameter as evidenced by scanning electron micrographs provided by Evonik [41]. However, in aqueous dispersion prepared in our laboratory, these nanocrystallites form – still relatively small – aggregates having a mean size of 50–60 nm (Figure 3 and Figure 4, red curves).

The particle size distribution of nanoTiO_2_ in the absence of destabilizing ions is relatively narrow and nearly harmonic (mean aggregate size: 55 nm) as shown in Figure 3 and Figure 4. In the presence of 0.1% NaCl, the loss of stability is indicated by the increase of the mean size to 3150 nm two hours after addition. When CMBCD-P is adsorbed onto the nanoTiO_2_, only partial aggregation was observed. A bimodal distribution was obtained. The smaller sized fraction (71% of the total sample) had a mean size of 90 nm, while the larger sized fraction (29% of the total sample) had a mean size of 1060 nm two hours after addition. As the photocatalytic degradation experiments were carried out for 60 min even this partial stabilizing effect of the polymer seemed to be enough for keeping the catalytic efficiency of the nanocatalyst.

In tap water, the stability of nanoTiO_2_ is similarly diminished as observed for 0.1% NaCl. The mean particle size increased to 4880 nm. It is notable that CMBCD-P proved to be more effective in this medium than in 0.1% NaCl solution. The initial particle size distribution of nanoTiO_2_ was nearly preserved, the mean size increased only to 59 nm, however, the distribution profile became slightly wider and a shoulder peak at 168 nm appeared on the curve.

### Photocatalytic effect of the CMBCD-P stabilized nanoTiO_2_ dispersions

**Figure 5:**
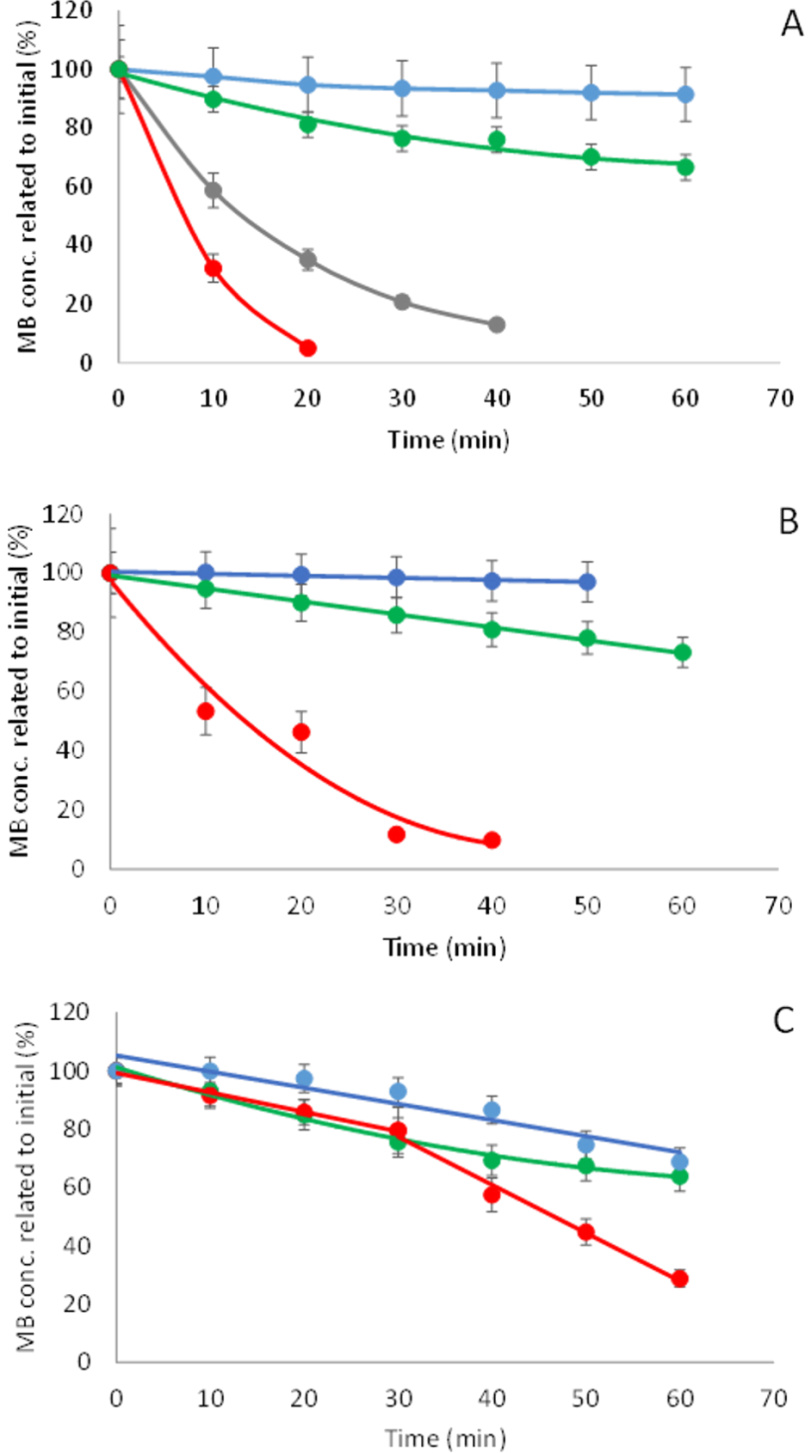
Photodegradation of MB in aqueous solutions: distilled water (A), 0.1% NaCl solution (B) and tap water (C) examining the dye itself (blue), in the presence of 1% CMBCD-P (green), of 0.02% nanoTiO_2_ (grey) and 0.02% nanoTiO_2_ stabilized by 1% CMBCD-P (red).

The photocatalytic effect of nanoTiO_2_ on the decomposition of two model contaminants: a dye (methylene blue, MB) and a drug (ibuprofen, IBR) was studied in various media (distilled water, NaCl solution and tap water) in the presence and absence of the polymer. The concentrations of the model compounds were measured as a function of time and the half-life time values were calculated to characterize the reaction rate of the degradation. For comparison, the decomposition of the model compounds was measured also without any additives and in the presence of CMBCD-P only. The results for MB are summarized in Figure 5 and Table 3.

**Table 3:** Half-life time (min) of photodegradation of MB in aqueous solutions in the absence and presence of CMBCD-P (1%), nanoTiO_2_ (0.02%) and nanoTiO_2_ (0.02%) stabilized by CMBCD-P (1%).

Medium	MB	MB + CMBCD-P	MB + nanoTiO_2_	MB + nanoTiO_2_ + CMBCD-P

Distilled water	495 ± 45	108 ± 11	13.5 ± 2.5	4.6 ± 0.5
NaCl solution (0.1%)	990 ± 70	136 ± 12	precipitation	11.3 ± 1.3
Tap water	107 ± 10	88.8 ± 7	precipitation	94.2 ± 0.5 (phase 1) 24.6 ± 3.6 (phase 2)

Interestingly, without catalyst MB was decomposed faster in tap water than in distilled water most probably due to the small concentration of iron present. Fe(III) and organic carboxylic acid, which coexist in natural environments, can set up a photo-Fenton system with H_2_O_2_ produced in situ generating OH radicals of high oxidizing capacity [42–43]. Nitrate could also produce OH radicals when absorbing UV light, but the low concentration of nitrate in tap water can insignificantly contribute to enhancing photodegradation.

The polymer itself showed some catalytic effect: the rate of degradation of MB was slightly increased in all the three media resulting in the shortest half-life time in tap water similarly to the dye alone.

The catalytic effect of nanoTiO_2_ itself could be measured only in distilled water, as both in NaCl solution and in tap water it was precipitated if not stabilized by CMBCD-P. In any media studied the reaction rate followed the order of: no additive < CMBCD-P < nanoTiO_2_ < nanoTiO_2_/CMBCD-P.

Applying the nanoTiO_2_ stabilized by the polymer synergistic effect was observed in distilled water. In NaCl solution the rate of decomposition was lower partly attributed to adsorption of chloride on TiO_2_ surface. There might be a competition between dyes and anions for the adsorption sites and the anions may modify the superficial properties of TiO_2_ [44]. The total concentration of inorganic ions in tap water is much lower than 0.1%, therefore this competition for the sorption sites is probably less pronounced even if we take into account that hydrogencarbonate, hydrogenphosphate and sulfate ions hinder the MB adsorption in a higher extent compared to chloride [44].

The nanoTiO_2_ stabilized by the polymer showed peculiar behavior in tap water: MB was decomposed in two distinct phases. In the first 30 min a slow degradation was observed (similar to that observed with the polymer only) and it was followed by fast decomposition (Figure 5 and Table 3). Similar two-stage degradation was observed for the photocatalytic decomposition of pharmaceutical residues, such as carbamazepine and ibuprofen in wastewater with TiO_2_ catalyst [42,45]. It was proved that the natural organic matter (NOM) which are the humic substances present in surface waters and tap water had an initial inhibiting effect: after the decomposition of NOM the drug degradation was enhanced [42,45]. The tap water (our test solution) also contained some dissolved organic components (1–2 mg/L) and this would explain our observation.

**Figure 6:**
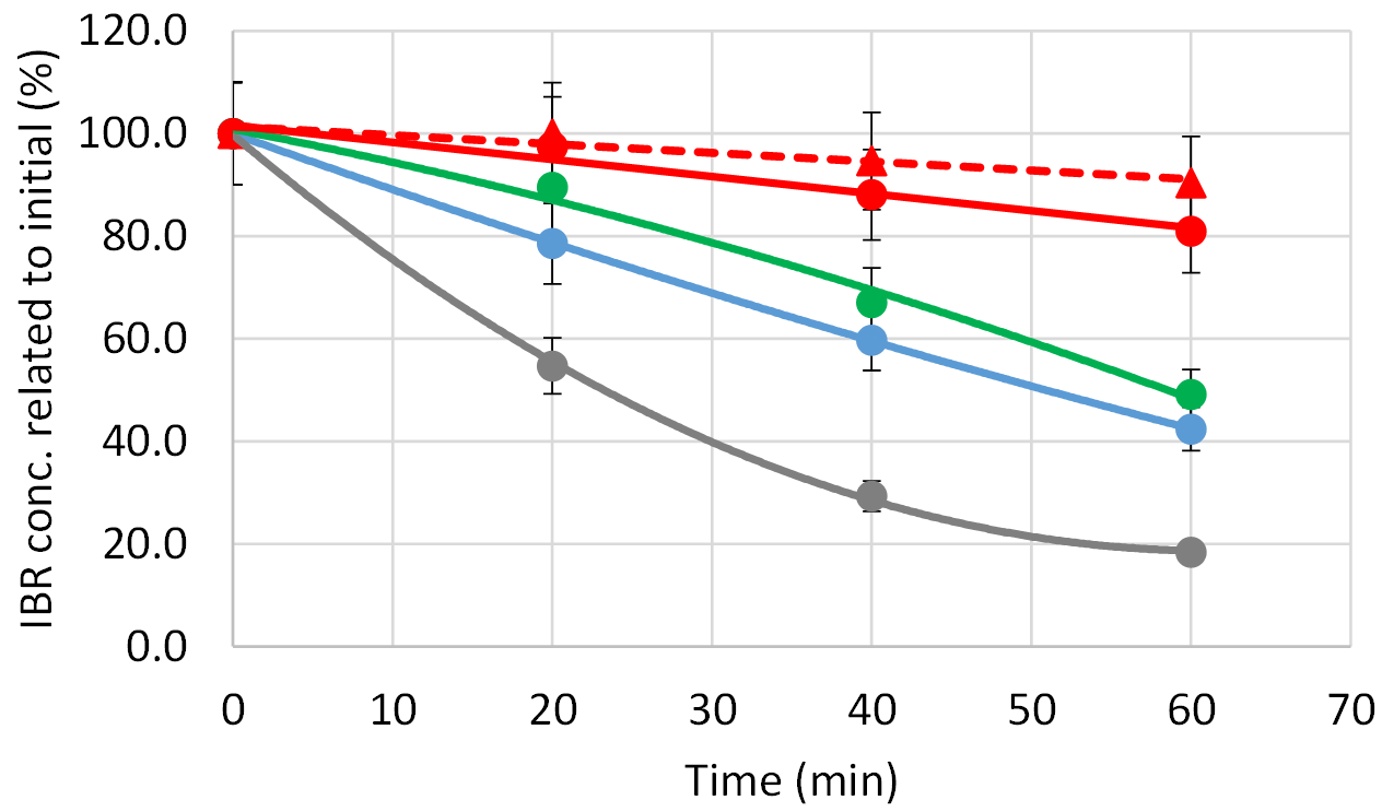
Photodegradation of IBR in distilled water examining the drug itself (blue circle), and in the presence of 1% CMBCD-P (green circle), of 0.02% nanoTiO_2_ (grey circle), 0.02% nanoTiO_2_ stabilized by 1% CMBCD-P (red circle), and 0.02% nanoTiO_2_ stabilized by 1% CMBCD-P in tap water (red triangle).

A different behavior was witnessed in the case of ibuprofen in our experiments. This drug was slowly decomposed in distilled water without any additives (Figure 6 and Table 4) and a slight enhancement in the degradation rate was obtained with nanoTiO_2_. In the presence of the polymer the rate of degradation decreased suggesting that the complex formation of ibuprofen has a protective effect. Using the CMBCD-P-stabilized nanoTiO_2_ as additive hardly any decrease in the drug concentration upon UV irradiation was measured especially in tap water.

**Table 4:** Half life time (min) of photodecomposition of IBR in distilled and tap water.

Medium	IBR	IBR + CMBCD-P	IBR + nanoTiO_2_	IBR + nanoTiO_2_ + CMBCD-P

Distilled water	31 ± 13	61 ± 18	25 ± 6	201 ± 56
Tap water	no data	no data	precipitation	331 ± 54

It is well-known that the complexation often has a protective effect on the included guest molecules [8]. Either catalysis or inhibition of the light-induced cleavage can occur depending on the conformation of the host–guest complex (if the light-sensitive part of the molecule is located within or outside of the cavity), on the complex association constant and on the concentration ratio of the guest to the CD. It was shown that the photodecomposition of MB was enhanced by stabilizing Ag/TiO_2_ nanoparticles with β-CD, but applying β-CD in excess the rate of degradation decreased dramatically [46]. The association constant of IBR/β-CD complex falls in the range of 10^3^–10^4^ M^−1^ [47–48], while that of MB/β-CD is less than 10^3^ M^−1^ [49–50]. According to NMR studies IBR is deeply included into the cavity [51], while MB is too large to be completely shielded by complexation. The too strong association between IBR and the cavities of CMBCD-P can be a possible reason of protection instead of catalytic decomposition in this process. Further experiments are needed to clarify why enhanced protection was observed when the polymer was present together with TiO_2_.

In order to check if the polymer itself is degraded similarly to the model pollutants as a control experiment, the CMBCD-P solution with and without nanoTiO_2_ was irradiated under identical conditions as the photodegradation of the pollutants. Size exclusion chromatography (HPLC) was used to characterize the composition of the polymer. This method enables to discriminate between the smaller and larger components and to monitor the degradation of the polymer. The chromatograms of the samples before and after irradiation match within the experimental error proving that no significant change in the polymer structure occurred upon UV light irradiation even in the presence of nanoTiO_2_ under the applied conditions (irradiation for 1 h). Lannoy et al. published the photodegradation of toluene in the presence of nanoTiO_2_ stabilized by native β-cyclodextrin and its random methylated derivative (BCD and RAMEB, respectively.) [36]. In the first hour 88% and 97% of the initial CD concentration was measured by phenolphthalein complexation, respectively, and 34% and 18% of these CDs were degraded in 5 hours. These results show that the substituted CDs are of higher stability compared to the unsubstituted one. The CMBCD-P is an epichlorohydrin-crosslinked polymer with the number of substituents/CD of approximately 10 (in RAMEB ≈12). Although the conditions of the photodegradation experiments published by Lannoy [36] are different from ours, the <5% degradation of CMBCD-P within 1 h seems to be in agreement with 3% degradation of RAMEB within the same time period. Further experiments are needed to reveal the fate of CMBCD-P during longer irradiation.

## Conclusion

We have successfully selected a potential stabilizing agent, carboxymethyl β-cyclodextrin polymer for improving the colloidal stability of nanosized TiO_2_. Dynamic light scattering measurements proved that the aggregation of TiO_2_ nanoparticles was hindered by the presence of this polymer additive. Studying its influence on the photocatalytic effect on nanosized TiO_2_ on one hand a synergistic photocatalytic effect was observed for photodecomposition of the methylene blue in tap water, on the other hand a protective effect of CMBCD-P was observed for the ibuprofen degradation. The efficiency of the CMBCD-P-stabilized nanosized TiO_2_ photocatalytic system will be studied on further model compounds selected among the emerging pollutants.

## Experimental

### Materials

Applying the procedure of CycloLab Ltd. Budapest Hungary a dispersion of 9.96% nanoTiO_2_ has been prepared from the Degussa-Evonik (Japan) titanium dioxide sample (Aeroxide P90 consisting of anatase 90% and rutile 10% with a specific surface area of 90–100 m^2^/g and mean particle size 14 nm**)**. This dispersion (pH was about 2) was diluted to 0.02% for the experiments. The cyclodextrins characterized by the degree of substitution (DS) in the case of monomers and by the CD content in the case of polymers (Table 5) are all CycloLab’s products. The DS and the CD content were determined by NMR as described earlier [52]. The molecular weights were determined by static light scattering as described by Puskás et al. [53]. The salts (NaCl, CaCl_2_, MgCl_2_, Na_2_CO_3_, Na_2_ SO_4_), NaOH and the model pollutants (methylene blue, MB and ibuprofen, IBR) are analytical grade chemicals purchased from Molar (Hungary).

**Table 5:** Cyclodextrins used during the experiments.

	Abbreviation	Characteristics	Average molecular weight (*M*_w_)^a^

hydroxypropyl-β-cyclodextrin	HPBCD-M	DS ≈4,2	1.38 kDa
hydroxypropyl-β-cyclodextrin polymer crosslinked with epichlorohydrin	HPBCD-P	CD content: ≈65–70%	90 kDa; 200 kDa; 300 kDa
carboxymethyl-β-cyclodextrin	CMBCD-M	DS ≈4	1.36 kDa
carboxymethyl-β-cyclodextrin-polymer crosslinked with epichlorohydrin	CMBCD-P	DS 2–3; CD content: ≈65–70%	33 kDa
quaternary ammonium β-cyclodextrin polymer crosslinked with epichlorohydrin	QABCD-P	DS ≈0.2 CD content: ≈65–70%	≈6 kDa

^a^For monomers it is calculated, for polymers data were obtained by static light scattering.

### Composition of tap water

The tap water in the district where the laboratory is located contains the following minerals according to the Budapest Potable Water Analysis [54]: Cl^−^ 21 mg/L, Na 11 mg/L, Ca ^2+^ 60 mg/L, SO_4_^2−^ 27 mg/L, HCO_3_^−^ 150 mg/L, NO_3_^−^ 5 mg/L, orthophosphate <5 mg/L. The electric conductivity is 480 μS/cm and pH 7.5. Total organic carbon content (TOC) 1–2 mg/L.

### Methods

#### Turbidity measurements

Turbidity of the nanoTiO_2_ dispersions was measured at 410 nm by Agilent 8453 spectrophotometer. The samples were measured in glass cuvettes of 1 cm.

#### Determinations of moisture content

The moisture content of the CMBCD-P has been determined using a 'Karl Fischer V20' type auto-titration device (Mettler Toledo).

#### Light scattering measurements

**Determination of average molecular weight by static light scattering:** The average molecular weight of the water-soluble polymers has been determined by static light scattering method using a Malvern Zetasizer Nano Series ZS device manufactured by Malvern Instruments Ltd., UK. The analysis was performed as described elsewhere [53].

**Particle size analysis by dynamic light scattering:** Determination of the particle size distribution of the nanoTiO_2_ particles and aggregates was performed by dynamic light scattering method (also known as photon correlation spectroscopy) using a Malvern Zetasizer Nano ZS instrument using a He-Ne laser of 4 mW power and 633 nm wavelength and avalanche photodiode detector. Triplicate measurements were carried out for all samples, each averaged of at least 10 runs at 25 °C. The volume size distribution was utilized for aggregate state analysis.

#### Photodegradation experiments

The degradation of methylene blue and ibuprofen was studied using an optical bench with medium pressure mercury-vapor lamp of 200 W (Tunsgram, Hungary) emitting mainly in the UV-A range. The light was focused by an optical lens on the quartz cuvette containing the sample. The concentration of MB and IBR was measured by spectrophotometry and by HPLC, respectively. The presence of polymer did not affect the spectrophotometric determination. The concentration of MB and IBR solutions was set to 20 and 50 μM, respectively before irradiation.

The half-life time was calculated postulating first order rate kinetics. Representing the concentration of MB or IBR in natural logarithmic scale as a function of time linear relationships were obtained proving the first order kinetics of degradation. The regression coefficients were in the range of 0.98–1.00. Three parallel measurements were evaluated.

#### Determination of methylene blue concentration

The concentration of the dye was measured by spectrophotometry using Agilent 8453 spectrophotometer at λ = 664 nm using 750 nm as reference.

#### Determination of ibuprofen concentration by HPLC

An Agilent 1100 HPLC system equipped with a diode array detector (254 nm) was used with a Waters ODS C18 (250 mm × 4.6 mm, 5 μm) analytical column and elution with 45% acetonitrile and 0.05% formic acid in water at a flow rate of 1.0 mL/min. The column temperature was set to 40 °C. The samples were diluted with 50% acetonitrile in 1:1 ratio before injecting 5 μL.

#### Characterization of the molecular weight distribution by HPLC

The same equipment was used with refractive index detector and TSK gel G2000SW silicagel based column (TosoHaas) (7.5 × 300 mm, 10 µm (molecular weight ranges: 1–30 kDa)), and guard column (7.5 × 75 mm) for size exclusion chromatography of CMBCD-P. The mobile phase (water, pH adjusted to 2.7 with cc. H_3_PO_4_) was eluted with 1 mL/min. The temperature of the column and of the RI detector was adjusted to 30 °C and 40 °C, respectively. The samples containing 10 mg/mL CMBCD-P were diluted to 5 mg/mL with the mobile phase and 20 µL was injected.
